# Nutrition in City Ecosystems (NICE): Protocol of a multi-sectoral development project to improve food and nutrition security of secondary city populations in Bangladesh, Kenya and Rwanda

**DOI:** 10.3389/fpubh.2023.1081535

**Published:** 2023-02-02

**Authors:** Cornelia Speich, Tanja Barth-Jaeggi, Capucine Musard, Cassien Havugimana, Charles Nwokoro, Elvis Gakuba, Farhad Zamil, Florence Sécula, Carmen Thönnissen, Johan Six, Klaus Kraemer, Kesso Gabrielle van Zutphen, Martijn Sonnevelt, Puja P. Tshering, Séverine Erismann, Sophie van den Berg, Simon Winter, Victoria Johnson-Chadwick, Marnie Pannatier, Breda Gavin-Smith, Dominique Barjolle, Helen Prytherch

**Affiliations:** ^1^Swiss Tropical and Public Health Institute, Allschwil, Switzerland; ^2^University of Basel, Basel, Switzerland; ^3^Swiss Tropical and Public Health Institute, Kigali office, Kigali, Rwanda; ^4^Syngenta Foundation for Sustainable Agriculture, Basel, Switzerland; ^5^Sustainable Agroecosystems Group, Institute for Agricultural Sciences, ETH Zürich, Zürich, Switzerland; ^6^Sight and Life, Kigali, Rwanda; ^7^Syngenta Foundation for Sustainable Agriculture, Dhaka, Bangladesh; ^8^Swiss Development and Cooperation (SDC) Global Food Security Programme, Berne, Switzerland; ^9^Sight and Life, Kaiseraugst, Switzerland; ^10^Johns Hopkins Bloomberg School of Public Health, Baltimore, MD, United States; ^11^World Food Systems Center, ETH Zürich, Zürich, Switzerland

**Keywords:** nutrition, diverse diets, secondary cities, food systems governance, agroecology, farmers' hubs, demand-side intervention

## Abstract

**Background:**

Secondary cities tend to be better linked with local food systems than primate cities, acting as important platforms to trade agricultural produce with rural surrounding. COVID-19, conflicts and climate change continue to expose inefficiencies in food systems and have further exacerbated malnutrition, calling for substantial food systems transformations. However, tackling current food systems' challenges requires new approaches to ensure food and nutrition security. Nutritious and agroecologically produced food offer the potential to transform food systems by improving diets and alleviating pressure on the environment, as well as by creating jobs and reducing poverty. This paper describes the design of a project by a Swiss public-private consortium to improve food and nutrition security and to reduce poverty in city ecosystems in six secondary cities in Bangladesh, Kenya and Rwanda through governance/policy and supply and demand side interventions.

**Methods:**

The Nutrition in City Ecosystems (NICE) project promotes well-balanced nutrition for city populations through interdisciplinary agricultural, food, and health sector collaborations along city-specific value chains. Adopting a transdiciplinary systems approach, the main interventions of NICE are (i) advocacy and policy dialogue, (ii) building of decentralized institutional capacity in multi-sectoral collaborations, (iii) support of data-driven planning, coordination and resource mobilization, (iv) anchoring of innovations and new approaches in city-level partnerships, (v) capacity building in the agricultural, retail, health and education sectors, as well as (vi) evidence generation from putting policies into practice at the local level. NICE is coordinated by in-country partners and local offices of the Swiss public-private consortium partners.

**Discussion:**

The NICE project seeks to contribute to urban food system resilience and enhanced sustainable nutrition for city populations by (A) strengthening urban governance structures involving key stakeholders including women and youth, (B) generating income for producers along the supply chain, (C) triggering change in producers' and consumers' behavior such that nutritious and agroecologically produced foods are both in demand as well as available and affordable in urban markets, and (D) allowing a scale up of successful approaches to other national and international cities and city networks.

## 1. Introduction

Sixty-eight percent of the world's population will live in urban areas by 2050, and around 90% of this increase will occur in small cities and/or towns of Africa and Asia (1). Small cities and towns are also the areas where the majority of the world's poor live today (2). Degradation of natural resources and pollution are often going along with rapid and unplanned urbanization. Urbanization costs also arise from the wasteful way in which many city food systems operate, including the overuse of fertilizers, excessive use of antibiotics for animal growth and untreated human waste (3). With more than 720 million people suffering from hunger, 149 million children under 5 years of age stunted and over 2.3 billion people not having regular access to sufficient, safe, and nutritious food (4), radical transformation of today's food systems is required to address urgent challenges of food security and nutrition. Issues of food security and insufficient nutrition not only lead to undernutrition and micronutrient deficiency but also foster overweight and obesity in many urban areas (4).

Urban food systems have impacts beyond just food, and their reach extends beyond just urban and peri-urban areas (2). Effective governance of urban food systems making use of multisectoral collaboration is a first step toward food systems transformation and tackling malnutrition issues (2). However, lack of articulation on nutrition outcomes in relevant urban policies and strategies, weak coordination mechanisms among stakeholders acting in food systems, lack of relevant institutional leadership, and lack of monitoring systems still often persist in many urban areas, and especially in the fast-growing secondary cities of Asia and Africa (2). Nutrition and food systems are multi-sectoral by nature, requiring expertise from agriculture, (public) health, finance, social affairs, education and many more (5). Based on principles of participation, ownership, and commitment, mutual trust and collaboration, participatory processes, and system approaches contribute to beneficial prioritizations, leverage synergies and improve the likelihood of success and sustainability of implementations (5). Discussions between municipal government and informal food sector associations such as e.g. consumer groups, farmer cooperatives (unions), civil societies etc. have been shown to importantly contribute to designing actions to improve nutrition and livelihoods, create jobs, reduce poverty and improve food and nutrition security for a large segment of the urban population (6). Similarly, it has previously been shown that women empowerment encouraging spousal discussions about farming contributes to increases in dietary diversity and increased nutrition practices (7). Women and also youth often play a key, but under-recognized and often informal, role in food systems e.g. in production, processing, and selling at markets and food shops. However, their participation in decision-making is often low and they only have limited opportunities to influence food systems. Thus, to get fair benefits from a food system that largely depends on them to function, women and youth are a priority population to be strengthened through food systems transformations (8).

Cities other than a country's largest city (primate city, often the capital) are named secondary cities and are generally better linked with local food systems than primate cities, acting as important platforms to trade agricultural produce with the rural surroundings (9). As such, secondary cities are important contributors to a reduction of rural poverty while primate citites lead in contributing to the country's economic development (9). In order for consumers in fast growing secondary cities to change their food consumption behavior toward improved diets and more sustainable food systems, nutritious and agroecologically produced food need to be available, accessible, and affordable. Containing ecological as well as social components focused on empowering the local context, agroecology may serve as the key overarching concept for sustainable food systems transformation (10). Increased proximity and connectivity between consumers and producers can reduce the risk of food contamination and maintain food integrity compared with long-distance travel (11). Furthermore, increased proximity between food production and food consumption in secondary cities' contexts can allow producers to earn a higher share of revenue and increase their margins due to lower investment e.g. into transport (11).

Particularly in the urban setting, many people currently experience a pronounced shift away from traditional staples such as rice, millet or pulses toward more convenient and often high-processed foods such as pasta, bread, or high-sugar foods (12–18). This is a result of changes in lifestyles including, but not limited to, moving out of a farming household, different relative prices for food, and often increased income (12). Urban households living in poverty tend to spend a large proportion (in some countries up to 70%) of their income on food, making them particularly vulnerable to food price crises (19–21). By forcing households to substitute nutritious food such as fruits and vegetables, nuts and seeds or animal products with less nutritious, less expensive, and less nutrient-dense staples, food price volatility immediately affects diet quality (9). Food and nutrition literacy emphasizing the ability of individuals to learn adequate food use, still seem to be insufficient to overcome these socio-economic obstacles. Hence, a systems approach combining the tackling of all health, environmental, and socio-economic factors to malnutrition is needed (22). Fragmented market structures contributing to the establishment of informal arrangements (street traders, home-based small retail stores) which are often not regulated, add another layer of complexity on the city food system (9, 23, 24). Still, it is not only physical and economic access shaping food and nutrition outcomes in urban contexts (25), but the consideration of how households utilize food together with clean water and sanitation and health care to reach adequate diets and achieve nutritional wellbeing, was found to be another important component in shaping households' abilities to ensure food security and dietary quality in Kisumu, Kenya (26).

It is in this context that a multi-country and multi-stakeholder project entitled “The Nutrition in City Ecosystems (NICE)” was conceived and provided with key funding by the Swiss Agency for Development and Cooperation (SDC). A public-private consortium comprising the Swiss Tropical and Public Health Institute (Swiss TPH), ETH Zürich (Sustainable Agroecosystems Group and World Food Systems Center), Sight and Life (SAL), and the Syngenta Foundation for Sustainable Agriculture (SFSA) is now implementing and co-financing the project to contribute to healthy nutrition through sustainable, local food production and more diverse and healthy dietary choices in urban food systems.

With its holistic approach addressing several sectors and layers of food systems, the NICE project aims to cut across six out of the 17 Sustainable Development Goals (SDGs) (27), namely:

- SDG 2—Zero Hunger through uncovering dietary patterns and promoting nutritious local food.
- SDG 3—Good Health and Well-being through diversified, micronutrient-rich food and nutrition.
- SDG 5—Gender Equity through the focus on women and youth.
- SDG 11—Sustainable Cities and Communities through the focus on urban and peri-urban populations.
- SDG 12—Responsible Consumption and Production through the promotion of agroecological food production and consumption.
- SDG 17—Partnerships for the Goals through the project's multi-stakeholder partnerships.

## 2. Methods/design

In this paper we describe the mixed-methods methodology system approach that is being used throughout the NICE project in city populations in Bangladesh, Kenya, and Rwanda. After an inception phase of 6 months, the project started in August 2021 and project phase I is currently ongoing until June 2025.

### 2.1. Study objectives and hypotheses

In alignment with SDC's thematic focus on food systems, the NICE project's primary objective is to improve the food and nutrition security of city populations and to reduce poverty by increasing the demand and supply of healthy, diverse diets consisting of nutritious and agroecologically produced food.

We hypothesize that:

IF city governments establish multisectoral platforms for nutrition planning and resource mobilization, and implementation is participatory with women and youth-led initiatives;

IF local food supply chains, built on a selection of food produced with improved knowledge on good agroecological farming practices, and supported by social business models along the value chain, are linked to urban markets;

IF knowledge about the importance of all aspects of diet (types of food, diversity, agroecological aspects) is generated and disseminated to urban, peri-urban and local consumers and producers (leaving no-one behind);

and

IF evidence from the project are not only shared among the participating cities and countries but also disseminated more broadly;

THEN NICE will contribute to (i) an increased demand for and supply of nutritious and agroecologically produced food, (ii) an improved nutrition situation of the whole city region population, (iii) strengthened governance of city food systems and the position of women and youth therein; and (iv) impacts that trigger a snow-ball effect beyond participating cities and countries.

**Figure 1:**
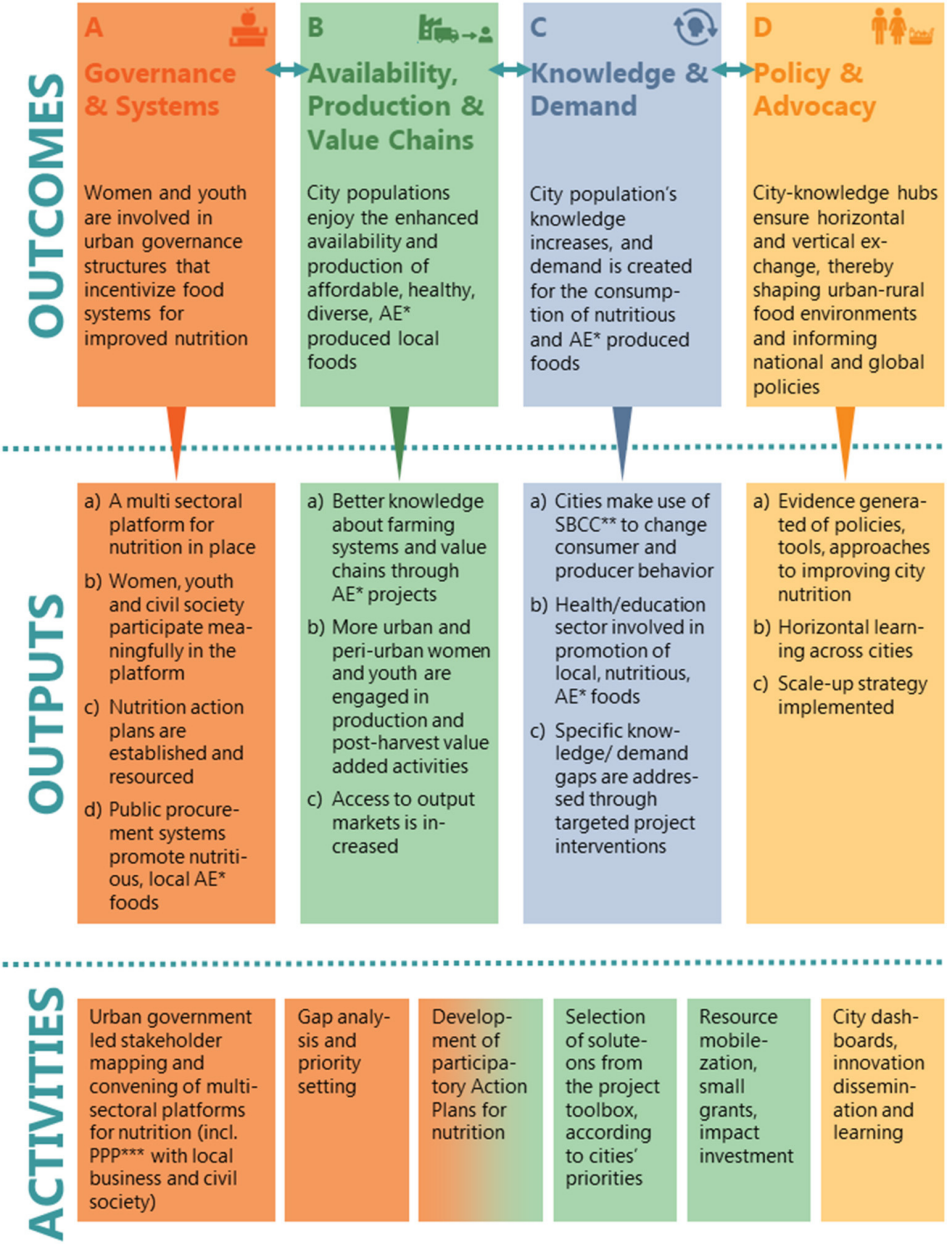
Theory of change of NICE with respective outcomes, outputs and underlying activities. In order to achieve its objectives to improve the food and nutrition security of city populations and to reduce poverty by increasing the demand and supply of healthy, diverse diets consisting of nutritious and agroecologically produced food, the NICE project works toward four outcomes **(A–D)** resulting from 13 outputs and their respective required activities. *AE, agroecology/agroecological/agroecologically; **SBCC, social behavior change communication; ***PPP, public-private partnership.

All project activities will foster four outcomes (A–D) *via* 13 clearly defined, expected outputs as presented in Figure 1 and will have a special focus on the inclusion of women and youth as priority populations for food systems transformation.

### 2.2. Project sites

In line with SDC's global perspective for this project, three countries have been selected from among SDC's focus countries for project implementation. Main criteria for country selection was the availability of a local office and network of one of the public-private consortium partners, capable to take on project management. In each of the three selected countries, two secondary front-runner cities (six cities in total) were chosen for the implementation of the NICE project. Selection of the cities was based on previous work experience of different members of the NICE consortium and the city's interest to be involved. The main target populations for the project activities are the socio-economically worst-off city populations living in poverty pockets with high rates of malnutrition as well as small holder farmers in the city food sheds, with a particular focus on women and youth. Through demand and affordability side interventions that can contribute to the availability, accessibility, and affordability of more diverse diets and thus improved nutrition, the nutritional status of all these populations should be improved.

In Rwanda, the selected secondary cities, Rubavu and Rusizi, are part of the Government's Second Economic Development and Poverty Reduction Strategy 2013–2018 promoting six cities to serve as additional country growth poles besides Kigali developing into a regional hub. In Bangladesh, the selected cities are Dinajpur and Rangpur. Bungoma and Busia are the selected cities in Kenya. The health and agricultural sectors are quite strongly devolved to county-level in Kenya, but the extent of decentralization is more mixed in Bangladesh and Rwanda.

The two Bangladeshi project cities Dinajpur and Rangpur are both located in the north-western part of Bangladesh. During consultation meetings with the city authorities in NICE's inception phase, the mayor of Dinajpur confirmed an estimated population size of ±300'000 for Dinajpur, with at least 45'000 of them living in one of the city's 69 slums where on average 8–9 households share one toilet (28). People in Dinajpur mainly belong to the ethnicities of Santal and Orao and Islam is their main religion while Bangla is their language. In terms of climate, Dinajpur faces few but heavy rains during the monsoon. Dinajpur City Context Analysis during NICE inception phase identified Dinajpur's economy to mainly depend on agriculture with a strong focus on rice production (28). Dinajpur has a governmental safety net program supporting people in need with food from the local storage depot. Furthermore, there are microcredit opportunities for women and youth and short-term (6–12 months) employment opportunities for unemployed youth at different government offices. As per the Rangpur City Context Analysis during NICE inception phase, Rangpur has a population of ±800'000 with at least 100'000 of them living in one of the city's 57 slums (29). People in Rangpur mainly belong to the ethnicities of Santal and Orao and Islam is their main religion. While Bangla is the formal language in the city, Rangpuri dialect is widely spoken in Rangpur's rural surroundings. Rangpur's climate is comparable to the one in Dinajpur; few but heavy rains during the monsoon. Assessed during NICE's inception phase, Rangpur city is a commercial hub that serves its surrounding districts. City dwellers are thus mostly involved in non-farming activities and Rangpur is one of the most important economic zones in Bangladesh, even though the city belongs to the most poverty-stricken regions of Bangladesh. Still, about 50–60% of agro-food products produced in the area are exported to the rest of the country. City dwellers usually purchase their food from local wet markets, where fish, rice, chicken, vegetables, and grocery items are available. Cereals, largely rice, are the main foods in Rangpur region (29). Also Rangpur has a governmental safety net program supporting people in need with food from the local storage depot.

The two Kenyan project cities, Bungoma and Busia, are both located in the western part of the country, close to the Ugandan border. Bungoma has a population of ±250'000 inhabitants (30) mostly belonging to the Luhya tribe (more precisely the Bukusu sub-tribe) with its own language, but Bungoma is becoming more and more cosmopolitan. Uncontrolled urban sprawl is gradually extending into prime agricultural land in the peri-urban areas of the town (31). In terms of climate, Bungoma faces a typically tropical climate with significant amounts of rainfall summing up in an average annual rainfall of 1,500 mm and an average annual temperature of 22.5°C (32). Maize covers 95% of the land under food crop production and 80% of the value of food crops produced annually in Bungoma county (33). Other crops are beans, sorghum, and millets as well as sugarcane, cotton, palm oil, coffee, tea and sunflower as cash crops (34). The main food processing value chains in Bungoma are maize into flour and animal feed, sugarcane into molasses and sugar, and coffee berries into coffee beans; most production is for local consumption (34). Busia has an estimated population of ±120'000 and rapidly growing informal settlements (35). The predominant ethnic groups in Busia town are Teso and Luhya with their own languages, while English and Kiswahili are widely spoken, and most inhabitants are Christians with also some Muslims especially in the urban center of the city (36). In terms of climate, Busia also faces a moisty tropical climate with a slightly higher amount of precipitation in the first half of the year compared to the second half summing up in an annual rainfall of 750–2,000 mm (37). Mean temperature is between 21 and 27°C in Busia (37). Besides agriculture and fishing, trade is another important economic activity in Busia (38). Agricultural production is mainly at a subsistence level. The main type of crops grown in Busia County include maize, cassava, finger millet, beans, sorghum, rice, sweet potato, cowpea, groundnuts, banana, green gram, sesame, soya beans, cotton, tobacco, sugarcane, oil palm, and pepper. The main value chains in the city-region are vegetables such as kales, cowpea, black nightshade, tomatoes, water melons, bananas, rabbit rearing, piggery and poultry rearing (39).

The two Rwandan project cities, Rusizi and Rubavu, are both located in the Western Province, the so-called food basket of Rwanda. Rubavu has a population of ±150'000 inhabitants (40). Main language in the area is Kinyarwanda and most people are Christians. In terms of climate, Rubavu faces an equatorial climate with an average temperature of 21.5°C as well as annual rainfalls of 1200–1300 mm fairly well distributed throughout the year except for the period of long dry season, which extends from June to mid-September (40). City Context Analysis during NICE inception phase highlighted Rubavu's high production volumes of potatoes, sweet potatoes, cassava, sorghum, maize, beans, vegetables, and fruits (mangoes and passion fruit) for subsistence and export to other regions of the country and beyond country's border to the Democratic Republic of Congo as well as of cash crops such as coffee, tea, and pyrethrum (41). Rubavu's economy is strongly dependent on cross border trade with Goma town in the Democratic Republic of Congo where 25% of Rubavu's population works. The tourism sector also fosters the economic development in the city and Rubavu is prominently mentioned in Rwanda's Tourism Policy (42), leading to a generally positive business environment in Rubavu. Less than 50% of the population are engaged in agricultural work in Rubavu, but just behind Kigali, Rubavu has the second most informal settlements among Rwandan cities, about 190 ha of the urban area are currently unplanned. Rusizi has a population of ±70'000 inhabitants (40). As for Rubavu, main language in the area is Kinyarwanda and most people are Christians. In terms of climate, Rusizi has an average temperature of 25°C, with hottest month being July. The average annual rainfall is 1200–1300 mm, fairly well distributed throughout the year except for the period of long dry season which extends from June to mid-September (40). City Context Analysis during NICE inception phase listed trade as another important economic activity besides agriculture, fishing, and forestry in Rusizi because the district shares borders with both the Democratic Republic of Congo and Burundi (40). Still, 57% of Rusizi's workforce are engaged in agriculture and 45% of Rusizi's population is categorized as poor or extreme-poor as per the Rwandan categorization system. Crops produced in the city include cassava, banana, sorghum, and peas. Other popular crops in Rusizi are avocadoes and French beans (41). In Rwanda, the socio-economically least well-off citizens are entitled to free health insurance while the wealthiest are paying premiums of USD $8 per adult per year (41).

As re-confirmed during City Context Analyses in the inception phase of the project, city-level nutrition data are scarce for all the selected cities, but Table 1 provides an overview on the most important nutrition indicators in the general urban context in the selected countries.

**Table 1:** Selected nutrition indicators to contribute to the big picture of cities in which the NICE project is implemented.

	**Bangladesh**	**Kenya**	**Rwanda**
< 5 y stunting prevalence (%)^a^	26.3 [2019]	20.0 [2014]	19.8 [2020]
5–19 y female overweight prevalence (%)^b^	8.7 [2016]	16.2 [2016]	16.9 [2016]
18+ female overweight prevalence (%)^b^	22.2 [2016]	34.3 [2016]	33.5 [2016]
Prevalence of infants with low birth weight (%)^c^	27.8 [2015]	11.5 [2015]	7.9 [2015]

While nutrition data for specific cities are scarce, the Global Nutrition Report's Country Profiles (66) provide a brief overview about national nutrition situations, with data on stunting prevalence disaggregated for the urban context only. Data in brackets indicating year of data collection.

^a^UNICEF/WHO/World Bank. Joint Child Malnutrition Estimates Expanded Database: Stunting, Wasting and Overweight. Published online July 2020. Available at: https://data.unicef.org/resources/dataset/malnutrition-data.

^b^NCD Risk Factor Collaboration. Values for 2000 to 2016 Published online http://ncdrisc.org/data-downloads.html.

^c^UNICEF/WHO. Low birthweight estimates. Published online 2019. Available at: https://data.unicef.org/topic/nutrition/low-birthweight.

### 2.3. Project design

The NICE project follows a context-sensitive / system approach focusing on governance and acting through facilitation and leveraging of local stakeholder activities in close partnership with the respective city authorities. While nutrition for city populations is improved through participatory, agricultural, food and health sector collaborations along city-specific value chains, interventions may differ among the participating secondary cities based on the food system opportunities and bottlenecks each city prioritizes; nevertheless expected outcomes and outputs of the project remain fixed (Figure 1). The main overarching interventions of NICE are thus (i) advocacy and policy dialogue, (ii) building of decentralized institutional capacity in multi-sectoral collaborations, (iii) support of data-driven planning, coordination and resource mobilization, (iv) anchoring of innovations and new approaches in city-level partnerships, (v) capacity building in the agricultural, retail, health and education sectors, as well as (vi) evidence generation from putting policies into practice at the local level, all around the four main project outcomes (Figure 1).

In Project Outcome 1, city authorities are supported to better understand the dynamics of their respective food system. With technical support, cities will build participatory mechanisms in the form of functional, multisectoral food systems platforms for improved coordination among several food systems stakeholders. These functional multisectoral food systems platforms including not only governmental organizations but also the private sector and civil society then aim to contribute to data-driven strategic planning and resourcing and make city food systems more responsive to local ecological conditions and nutritional needs of its population in an inclusive manner (43, 44). The example of Brazil, which used to be an exemplary case of governmental support for agroecology but then was completely wiped out by a change in political leadership (45), illustrates the importance of strong and resilient/robust multisectoral, local level food systems ownership.

In Project Outcome 2, availability, accessibility and affordability of nutritious and agroecologically produced food shall be addressed through implementation and strengthening of farmers' hubs. Under the concept of farmers' hubs—an inclusive business model developed by SFSA—commercial one-stop service platforms create small holder farmers' access to quality inputs, agricultural machines, markets, finance and knowledge, ensuring them fair prices and assistance for increased farm productivity (46). Challenges of the agri-food chain including farming systems, food safety, supply chain (e.g., regarding intermediaries engaged in trading), and post-harvest handling shall be addressed (e.g., in the form of trainings and study tours) in line with the complex and dynamic concept of agroecology defined by the framework of the High Level Panel of Experts (HLPE) of the Committee on World Food Security and Nutrition (47). The framework bases on a comprehensive set of 13 agroecological principles as presented in Table 2. Value chains which the NICE project should focus on will be selected in a collaborative and participatory approach focusing on (i) government buy-in, (ii) nutrition-improvement potential, (iii) production feasibility, (iv) market potential, (v) income generation potential, (vi) agroecology potential and (vii) consumer buy-in. The UN Food and Agriculture Organization's (FAO) Self-evaluation and Holistic Assessment of climate Resilience of farmers and Pastoralists (SHARP) tool will be adapted to the needs of the project to understand the agroecological status of each value chain, allowing a thorough gap assessment and challenges identification in the farming system (48, 49). After prioritization of the main value chain-related challenges, project interventions will be decided in consultations with key stakeholders following the International Fund for Agriculture Development (IFAD)'s guide for project design in nutrition-sensitive value chains (48).

**Table 2:** Comprehensive set of 13 agroecological principles as per the High Level Panel of Experts of the Committee on World Food Security and Nutrition's framework of agroecology (47).

**1. To improve resource efficiency**
a. Recycling
b. Input reduction
**2. To strengthen resilience**
c. Soil health
d. Animal health
e. Biodiversity
f. Synergy
g. Economic diversification
**3. To secure social equity / responsibility**
h. Co-creation of knowledge
i. Social values and diets
j. Fairness
k. Connectivity
l. Land and natural resource governance
m. Participation

In Project Outcome 3, demand for nutritious and agroecologically produced food should be fostered through social behavior change communication influencing evidence-based decision-making by local actors on food production and consumption behaviors. Social behavior change communication increasing the nutrition literacy and thus the demand for nutritious and agroecologically produced food will include a range of media campaigns and social marketing interventions informed by evidence from a qualitative formative research through stakeholder interviews, in-home observations and group discussions. Consumers should become participants rather than just “beneficiaries” of food system transformation and the project's focus will be on nutritious and agroecologically produced food across selected city food regions emphasizing on ensuring access for women, youth and people living in informal settlements.

**Figure 2:**
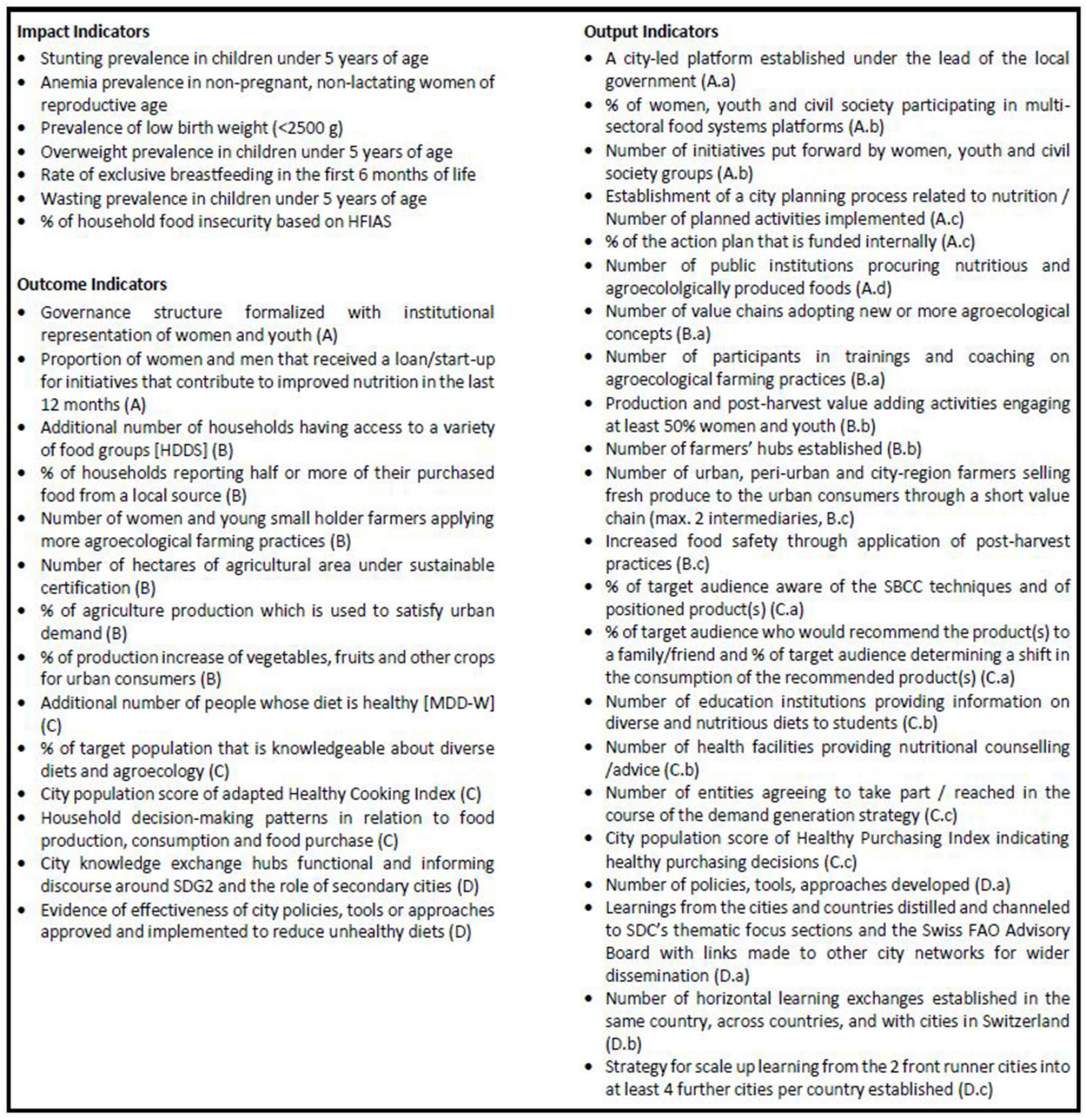
Monitoring and Evaluation indicators of NICE. A comprehensive, results-oriented M&E system based on a logframe supports the steering of the project and the generation of evidence to contribute to policy dialogue and wider learning *via* impact, outcomes (A–D referring to outcomes in Figure 1), and output indicators (A.a–D.c referring to outputs in Figure 1). HFIAS, Household Food Insecurity Access Scale; HDDS, Household Dietary Diversity Score; MDD-W, Minimum Dietary Diversity for Women.

Finally, in Project Outcome 4, robust monitoring and evaluation (M&E) of the whole NICE project is ensured and lessons learned are recorded to be shared within and across countries. Data on urban population-specific food systems indicators are essential to guide city authorities' decision-making and to monitor change: As an example, egg hub models, where eggs are produced safer and more efficiently through collaboration, and support low market prices, are a true success story of social business implementation by SAL (50). Hence, they acted as an inspiration for the systematic approach in NICE (50): Increased egg production to lower market prices not only made eggs more accessible for those most in need of nutritious food—women and children—but also raised the incomes of smallholder (women) farmers in SAL's experience (50). Food systems data collected in the NICE project will be made publicly available in due time through (peer-reviewed) publications, local outreach documents such as case studies, good practices or technical briefs, and on city-owned online urban food system fora to further inform food systems transformation. Food systems data to be collected in the NICE project include baseline and endline data on NICE's impact and outcome indicators (Figure 2), data of the formative research on consumer and farmer behavior to build the evidence for social marketing and agroecology interventions as well as qualitative findings from food systems governance experience.

### 2.4. Project governance

City-level partnerships are at the core of NICE's context-sensitive / system approach and facilitation is a key component of the project. With assistance from the NICE project, city authorities (mainly from the departments of health and agriculture, but also departments of development, social welfare, education, finance etc.) and other food system stakeholders (farmers' cooperatives, local small and medium size enterprises, women and youth associations, nutrition counselors and primary health care points, local NGOs etc.) are leading the implementation of activities that support both overarching city-led priorities, as well as the project goals and outcomes. Innovations and new approaches, especially regarding agroecology and social behavior change communication, are foreseen to be anchored in the city-level partnerships.

**Figure 3:**
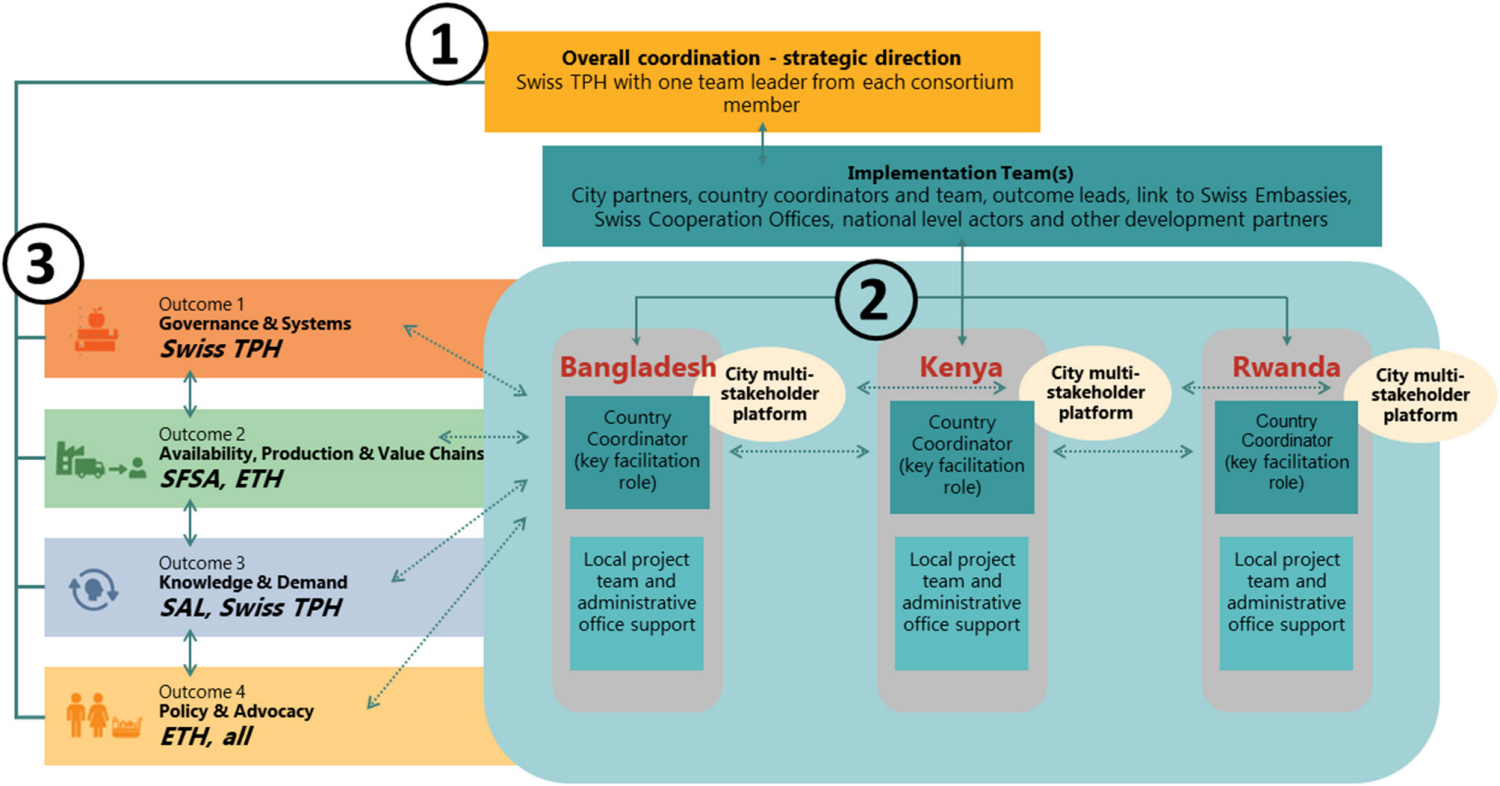
Overall set-up of NICE. NICE is set-up around 3 levels of governance: (1) overall coordination and steering of the project by a leadership board consisting of the team leader from Swiss TPH and one team leader from each consortium member; (2) country-led implementation through country-level project coordinators and their teams consisting of city-based coordinators as well as assisting staff; (3) cross-cutting technical support and quality assurance across cites and countries by global outcome teams bringing in the specific expertise of all four consortium partners. Swiss TPH, Swiss Tropical and Public Health Institute; SFSA, Syngenta Foundation for Sustainable Agriculture; ETH, Eidgenössische Hochschule Zürich; SAL, Sight and Life.

Local SFSA offices in Bangladesh and Kenya, and the SAL and Swiss TPH offices in Rwanda backstop project implementers on the ground. All project activities are managed across several levels (Figure 3). On a first level, there is overall coordination and steering of the project by a leadership board consisting of the project leader from Swiss TPH and one team mate from each consortium member. On a second level, city-led actions are facilitated by the country-level project coordinators and their teams consisting of city-based coordinators as well as assisting staff. On a third level, backstopping and crosscutting technical support across cities and countries are provided by the global outcome teams bringing in the specific expertise of all four consortium partners: As a prominent institute in global health and nutrition, with experience in working with local governments and expertise in systems strengthening, Swiss TPH is responsible for Outcome 1. SFSA with its farmers' hubs model and wide expertise in agriculture, agribusiness, value chains, and markets is backstopping Outcome 2, strongly supported by ETH Zurich with its deep knowledge on agroecology and implementing impactful supply side interventions and analyses to improve food security, income, and resilience. SAL, a global nutrition think tank, with a wide set of expertise in nutrition, behavior change, and brokering public-private partnerships, backstops Outcome 3 while Outcome 4 is backstopped by ETH Zurich with its Sustainable Agroecosystems Group and the World Food System Center globally recognized for their expertise in agriculture, agroecology, food systems, and city region resilience.

An Advisory Board of food systems, nutrition, agroecology and urbanization experts as well as country experts from policymaking, academia, and project partners including SDC, guides the strategic direction of the project by meeting twice a year to oversee study progress in an independent manner, giving feedback and making recommendations.

Finally, a comprehensive, results-oriented M&E system based on a logframe supports the steering of the project and the generation of evidence to contribute to policy dialogue and wider learning. Indicators for M&E are presented in Figure 2.

Baseline data on impact and outcome indicators have been collected through a baseline investigation by independent local academic partners (Bangladesh, Kenya) and Swiss TPH (Rwanda) from April to June 2021 in all the cities involved and will be published separately. These information guide the value-chain selection and the identification of future study beneficiaries (priority populations). Similarly, a respective endline investigation is planned for the end of the project to assess improvements. Data on output indicators are generally collected on an on-going or bi-annual base by the country project management teams through focus group discussions and key informant interviews as well as respective observations and document collections. Furthermore, latest at the midpoint of the project, an internally arranged review will be conducted to confirm the relevance, efficiency, and effectiveness of the interventions, to gather project beneficiaries' experiences, and examine progress with the scale-up strategy.

A conflict-sensitive program management approach is implemented for planning, facilitating, and evaluating project interventions as the project has the potential to disrupt the status quo, potentially triggering conflict between local partners.

### 2.5. Data management and ethics

All project data will be collected electronically in this study. As agreed in any study protocols submitted for ethical clearance in the NICE project, raw data will be uploaded onto encrypted, secure servers of the Swiss headquarters of the respective academic partners, and will be rapidly curated, anonymized and cleaned before storage. All data will always be deleted from devices used in the field after upload to the main respective server. Data cleaning will be undertaken in respective statistical programs such as STATA or R, and various checks will be run on quantitative data to check for outliers, inconsistencies and potential mistakes.

Local authorities will be closely involved in all activities in their municipalities, or wider districts, including in the development of annual workplans and sharing of budgets. Informed by the fact that different malnutrition problems in urban centers tend to be clustered by residential neighborhoods, areas that have high rates of malnutrition are identified and particularly supported for and by the different project interventions, in close consultation with the local authorities.

Ethical clearance for any data collection and surveys will be carried out as requested by national bodies and regulations, especially given that some data collection will involve vulnerable population groups, and include any personal data and anthropometric measurements. The NICE project will work with local academic partners and involve them in dissemination of findings. Survey results will always be fed back to local authorities and the involved communities in the cities concerned.

An assessment of the main contextual, programmatic and institutional risks of the NICE project as well as an in-depth consultation process have been carried out during project preparation. The consortium partners are well networked in all three countries and specifically with the local municipalities in all the cities.

## 3. Discussion

Suboptimal diet is responsible that one-third of the world's population suffers from malnutrition (4). Current food systems cannot guarantee sustainable availability, accessibility and affordability of nutritious and agroecologically produced food for all city dwellers in many urban areas (4, 51). Man-made conflicts, climate change and COVID-19 are further accentuating the burden of malnutrition and food insecurity, and the global community, therefore, recognizes an urgent need for food systems transformation toward more sustainable ways of producing and consuming food (52). By signing initiatives such as the Milan Urban Food Policy Pact or the C40 Cities Climate Leadership Group, many cities around the world already acknowledge the strong potential cities and urban regions can play for successful implementation of beneficial food systems transformations: By participating in large multisectoral networks with common aims and objectives, cities support each other through peer-to-peer exchange and direct technical assistance as well as knowledge sharing and efforts management and take their responsibility to integrate sustainable food systems into social, economic and environment policies, programs and initiatives (53, 54). The recent United Nations (UN) 2021 Food Systems Summit combined crucial elements of food safety, nutrition, poverty and inequalities in the context of climate and environmental change to ensure that all people have access to a safe and nutritious diet (55, 56). The UN 2021 Food Systems Summit thus aimed to catalyze a shift in consumer behavior that will create and build demand for sustainably produced agri-food products (55, 56). The NICE project is directly in line with Action Track 1 and Action Track 2 of the UN 2021 Food Systems Summit.

Sustainability is a key requirement of the NICE project, particularly fostered through interventions in the field of agroecology and social businesses (47, 57). Agroecology, by promoting sustainable farming practices in different categories has increasingly gained scientific and policy recognition as a way to address environmental and social issues within food systems (58). With investments in systems research, innovation, capacity building, market linkages, and the realization of fair prices, a huge potential can be exploited from agroecology to transform food systems in low-income countries (59). There is also a body of evidence on how women's participation in agroecological networks (especially in short supply chains) helped them to lift themselves out of violent situations of isolation and to affirm their own identity and knowledge (60, 61). Social businesses are another promising approach for improved sustainability and women engagement (57): Rural employment and entrepreneurship are key potential drivers of economic growth, as well as being vital for food and nutrition security. Acting as aggregators for input buying and output sales, as well as providing good agricultural practice know-how and machinery, Farmers' Hubs are promising examples for the social business model and are particularly strengthened by the NICE project. Young people want opportunities and incentives, the chance to learn new skills, use new tools and earn a decent income in new markets. Farmers' Hubs are mostly driven by young entrepreneurs linking farmers in their communities to modern agricultural technologies and practices.

Work in nutrition and food systems is multisectoral by nature as it requires expertise from agriculture, public health, nutrition, education and beyond (5). Tight collaboration between supply and demand side as well as food systems governance guarantees affordable availability and accessibility of nutritious and agroecologically produced products, and nutrition-literate consumers' demand. Through widely-disseminated, well-timed and designed social behavior change communications on several media, nutrition literacy and maturity of city populations are improved, influencing city populations' dietary patterns.

Literature has shown that by involving a broad base of stakeholders and basing the policy and planning processes on principles of participation, ownership, commitment, mutual trust, and collaboration, municipal authorities are more likely to develop policies and programs that meet the needs of both the municipality and its constituents, and are thus more inclusive and successful in implementation (5, 62). Dubbeling et al. (2010) summarized the benefits of applying a participatory and multisectoral approach in transformation processes as follows: (i) More participatory governance and encouraged public-private partnerships help overcome distrust, and bridge the gap between citizen groups and the local government; (ii) A better understanding of priority issues and the needs of different food systems stakeholders empower respective quality analyses and decision-making; (iii) Enhanced acceptance and ownership of the transitions improve likelihood of success and sustainability of implementation, and (iv) Problem-solving and political lobbying capacities of the participating institutions are strengthened, and citizen's groups are empowered (5, 63, 64). Still, participatory, multisectoral approaches also have their challenges that need to be tackled, including amongst others a higher time investment compared to conventional top-down approaches or the danger of undue increases in the influence of some stakeholders with higher capacity to actively participate in the process and to convince other stakeholders (5, 63, 65). Through continued awareness-raising and information dissemination among and toward multiple stakeholders feeling ownership for the local urban food system, the NICE project will contribute to institutionalization of more sustainable food systems providing affordable nutritious and agroecologically produced food to all city dwellers, even the ones most at risk for malnutrition due to cultural and socio-economic shortcomings. Active strengthening of the organizational, managerial, technical, and networking capacities of all food system stakeholders, particularly focusing on women and youth, is key for making transitioned food systems more inclusive. The prioritization of women and youth as important beneficiaries of improved food systems but also key actors within them, challenges current power imbalances and inequities in access to resources and decision-making.

Through its context-sensitive / system approach fostering human-centered, participatory, agricultural, food, and health sector collaborations, the NICE project will improve and transition food systems by (A) strengthening urban governance structures involving key stakeholders including women and youth, (B) generating income for the producers along the supply chain, (C) triggering change in producers' and consumers' behavior such that nutritious and agroecologically produced food are both in demand, available and affordable in urban markets, and (D) scaling up successful approaches to other cities within the countries, as well as internationally. By channeling experiences into national policies and exchanges, city-level and national level project ownership as well as social accountability are strengthened. The front-runner project cities in each country are expected to share their experiences and findings with four additional cities per country during this project phase. In a potential second phase of the project, the findings and interventions should also be transferred to other countries, focusing on an involvement of also francophone contexts, and more fragile contexts, potentially with links to humanitarian aid.

## Author contributions

CS supported the development of country-specific protocols, coordinates and monitors the study implementation, and drafted the manuscript. TB-J, KZ, and HP co-designed the project, supported the development of country-specific protocols, coordinate and monitor the study implementation, and drafted the manuscript. CM, SE, VJ-C, CT, and FS co-designed the project. CH, CN, EG, FZ, SB, and MP supported the development of country-specific protocols and coordinate and monitor the study implementation. JS, KK, MS, PT, SW, BG-S, and DB co-designed the project, supported the development of country-specific protocols, and coordinate and monitor the study implementation. All authors read and approved the final manuscript.
